# Treatment of COPD and COPD–heart failure comorbidity in primary care in different stages of the disease

**DOI:** 10.1017/S1463423620000079

**Published:** 2020-06-05

**Authors:** Pietro Pirina, Elisabetta Zinellu, Marco Martinetti, Claudia Spada, Barbara Piras, Claudia Collu, Alessandro Giuseppe Fois

**Affiliations:** 1 Department of Respiratory Diseases, Azienda Ospedaliero Universitaria, Sassari, Italy; 2 Respiratory Unit, Department of Medical, Surgical and Experimental Sciences, University of Sassari, Sassari, 07100 Italy; 3 General Practitioner, Carbonia, Italy

**Keywords:** COPD, HF, COPD severity, pharmacological treatment, primary care

## Abstract

**Background:**

Chronic obstructive pulmonary disease (COPD) is a chronic respiratory disease that may have a negative impact on both patients’ quality of life and survival. Patients with COPD frequently suffer from heart failure (HF), likely owing to several shared risk factors.

**Aim:**

To evaluate the differences in treatment of COPD with and without HF comorbidity according to COPD severity in the general practitioner setting.

**Methods:**

We conducted an observational, retrospective study using data obtained from the Italian Health Search Database, which collects information generated by the routine activity of general practitioners. The study sample included 225 patients with COPD, alone or combined with HF.

**Findings:**

It has been found that the prevalence of some comorbidities such as diabetes and HF significantly increases with the severity of COPD. Regarding pharmacological treatment, a reduction in the prescription of individually administered long-acting β 2-agonists (LABAs) and long-acting anticholinergics (LAMAs) has been observed with increasing severity of the disease. Moreover, an increase in the prescription of both the combination of the two bronchodilators (LABA + LAMA) and their association with inhaled corticosteroids has been observed with increasing severity of COPD. The prescription of β-blockers in patients with COPD suffering from HF comorbidity decreases from 100% in stage I to less than 50% in the other stages of COPD. This study shows that general practitioners do not follow the guidelines recommendations for the management of patients with COPD in the different stages of the disease, with and without HF comorbidity, as well as in the management of HF. Further efforts must be made to ensure adequate treatment for these patients.

## Background

Chronic obstructive pulmonary disease (COPD) is a chronic respiratory disease characterized by progressive airflow limitation that is not fully reversible, airways inflammation, and systemic effects that may have a negative impact on the patient’s prognosis, quality of life, and survival (Barnes, 2000; Decramer *et al.*, 2012). The impairment in forced expiratory volume in the first second (FEV1), the best measure to assess airway obstruction, defines four grades of severity (stage I: FEV1 ≥ 80% predicted; stage II: 50% ≤ FEV1 < 80% predicted; stage III: 30% ≤ FEV1 < 50% predicted; stage IV: FEV1 < 30% predicted) (Miravitlles *et al.*, 2017a; Vogelmeier *et al.*, 2017b). COPD entails a significant economic burden, including hospitalization, absence from work, and disability. Epidemiological evidence shows that it is currently the third leading cause of death in the world (Lozano *et al.*, 2012), and its mean prevalence in Europe in adults older than 40 years ranges from 10% to 14% (Blanco *et al.*, 2018). COPD is frequently associated with systemic manifestations and comorbid conditions (Fabbri *et al.*, 2008). In particular, COPD frequently coexists with heart failure (HF), and this association suggests common risk factors, such as cigarette smoking and advanced age (de Miguel Díez *et al.*, 2013). HF is a complex clinical syndrome with typical symptoms such as dyspnoea, fatigue, and edema, and objective signs of structural or functional abnormalities of the heart at rest (Hunt *et al.*, 2009). The prevalence of HF ranges from 1% to 12% according to data from different countries (Roger, 2013), and it has been reported as high as 41% in patients with COPD (Chen *et al.*, 2015). Since the association of COPD and HF has a worse prognosis than either condition alone (de Miguel Díez *et al.*, 2013), a combined approach in managing these comorbidities is needed.

Patients with emphysema and HF have been found to have a reduction in left ventricular filling, left ventricular stroke volume, and cardiac output, although left ventricular ejection fraction is generally preserved (O’Donnell and Parker, 2006). These cardiac disorders in patients with COPD may occur as a result of various mechanisms, including loss of pulmonary vascular bed, hypoxic pulmonary arterial vasoconstriction, pulmonary hyperinflation, and elevated intrathoracic pressure. Currently, it is not known whether the same cardiac disorders are also present in milder COPD patients (Barr *et al.*, 2010). A correct use of bronchodilators for COPD treatment reduces pulmonary hyperinflation while improving cardiac performance.

The treatment of all stages of COPD includes the use of inhaled long-acting β 2-agonists (LABAs) and long-acting anticholinergics (LAMAs), individually or in combination, in order to optimize bronchodilation, reduce pulmonary hyperinflation, and thus improve patient exercise tolerance. Inhaled corticosteroids (ICSs) are currently reserved for patients whose symptoms and exacerbations are not optimally controlled by bronchodilators administered in combination, in patients with elevated peripheral eosinophil count and in those with asthma–COPD overlap (Calverley *et al.*, 2017). Although bronchodilators may induce side effects such as tachyarrhythmias and hypokalemia, it is also true that both LABAs and LAMAs have not shown a significant increase in cardiovascular side effects or any increase in mortality compared to placebo in the most important randomized controlled trials (RCTs) (TORCH and UPLIFT/FLAME, respectively) performed in COPD patients in the last decades (Campo *et al.*, 2015).

β-blockers represent the first choice treatment in HF. With regard to this, cardioselective β-blockers have proven to be safe and effective in the treatment of patients with COPD–HF comorbidity. To date, there is a robust literature that shows that cardioselective β-blockers do not hold a significant impact on lung function decline in these patients, on the contrary they are associated with a better prognosis and reduction in mortality (Short *et al.*, 2011; Ni *et al.*, 2012).

In a previous study, we showed that during diagnostic workup, general practitioners acted differently with patients with COPD as opposed to patients with HF, with higher adherence to guidelines in the latter disease. This imbalance was confirmed even when the two diseases coexisted (Pirina *et al.*, 2017). In this study, we aimed to evaluate how general practitioners manage patients with COPD in different stages of disease severity, depending on the presence of HF comorbidity. This study will provide additional data on the management of these comorbidities with the purpose to improve treatment, survival, and quality of life for these patients.

## Methods

### Data collection

We conducted an observational, retrospective study using data obtained from the Health Search Database (HSD), which contains information on the activity of general practitioners. From this database, 50 530 files, corresponding to 50 530 patients, were analyzed for data extraction. The extraction of data was done between 1 July and 31 December 2016. Only patients who were treated continually between 1 January 2015 and 30 June 2016 were considered. International Classification of Diseases (ICD) codes (10th revision) were used to identify the diseases. In particular, J44 group and I50 group referred to COPD and HF, respectively (World Health Organization, 2016). A few comorbidities such as hypertension and diabetes were evaluated. Conversely, other cardiovascular diseases, such as ischemic heart disease or arrhythmias, were not considered for this study.

### Patients selection

The patients were selected based on the presence of a diagnosis of COPD identified by the ICD codes group J44. Among these patients, we considered only those who had performed a spirometry, which is essential to assign disease severity categories according to the Global Initiative for Chronic Obstructive Lung Disease (GOLD) strategy, 2017 updated (Vogelmeier *et al.*, 2017b). The HF comorbidity was evaluated by the identification of the ICD codes group I50. In addition, most patients had an instrumental finding: 95.4% of them had an electrocardiogram and 81.5% had an echocardiogram (EcoCG). The study sample included 225 patients with COPD, alone or combined with HF. We have analyzed clinical parameters, risk factors, and pharmacological treatment of the patients involved.

This study is a retrospective analysis of data already available in the HSD. The data were collected anonymously in accordance with the privacy laws. Therefore, an approval by an ethic committee was not requested.

### Statistical analysis

Quantitative variables are expressed as mean values (mean ± SD), while categorical variables are expressed as percentages. Student’s *t*-test for quantitative variables and chi square test for categorical variables were performed in order to evaluate the significance of observed differences regarding the clinical characteristic of COPD patients and the medical regimens prescribed based on disease severity. Outcomes with a *P*-value ≤ 0.05 are considered statistically significant.

## Results

**Figure 1.**
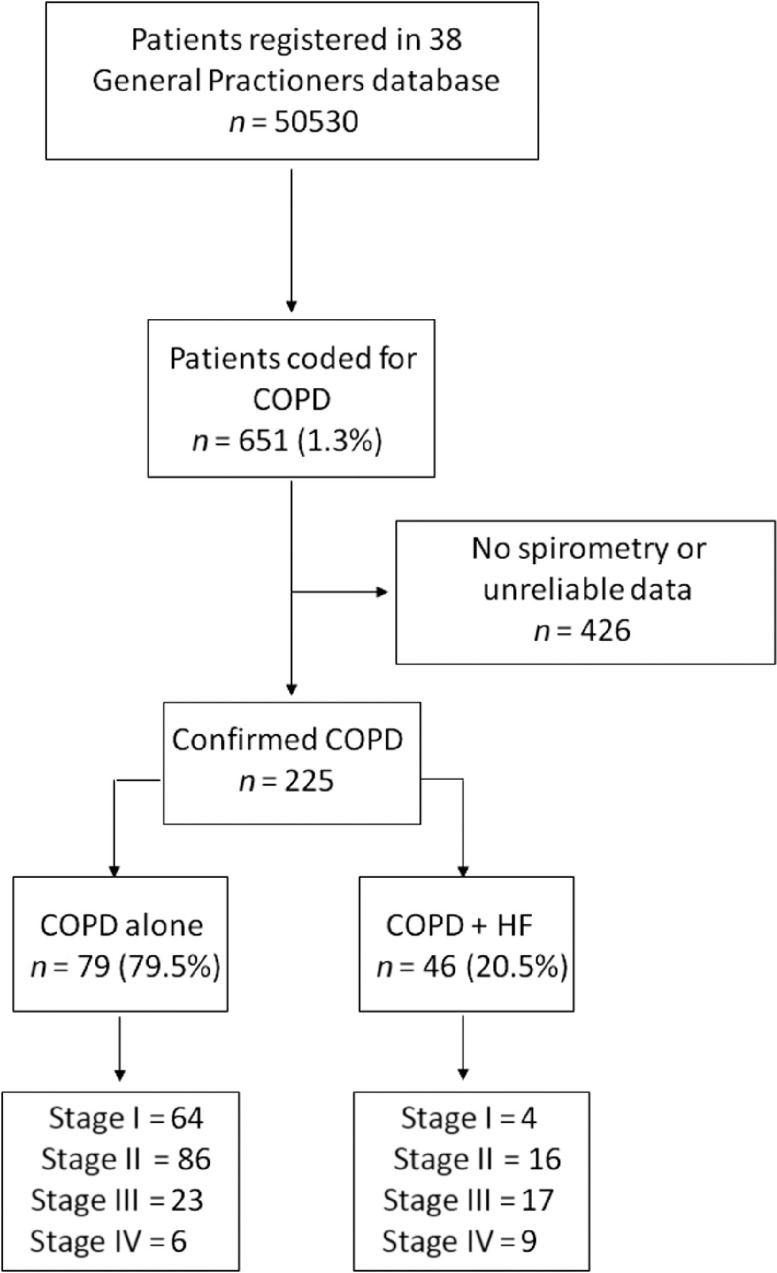
Diagram showing the patients selection

A total of 225 subjects diagnosed with COPD were included in this study. Of those, 179 subjects suffered from COPD alone, and 46 suffered from COPD and HF comorbidity (Figure 1).

Table 1 reports the clinical characteristics of these patients based on COPD disease severity. Other than the high prevalence of patients with a history of smoking exposure, this table shows an increase in mean age with increasing disease severity (67.8 ± 11 in stage I to 78.1 ± 11.9 in stage IV, *P* < 0.01) and an increase in some comorbidities, such as HF (5.9% in stage I to 60% in stage IV, *P* < 0.001), diabetes (8.8% in stage I to 33.3% in stage IV, *P* < 0.05), and hypertension (63.2% in stage I to 73.3% in stage IV, *P* = 0.66). Conversely, levels of total plasma cholesterol decreased with severity of COPD (stage III compared with stage I, *P* < 0.05), while the body mass index settled at the lower limits of normality in all stages.

**Table 1.** Clinical characteristics of chronic obstructive pulmonary disease patients according to disease severity

	GOLD I (*n* = 68)	GOLD II (*n* = 102)	GOLD III (*n* = 40)	GOLD IV (*n* = 15)
Age	67.8 ± 11	71.6 ± 10.3*	75.6 ± 10.7^***†^	78.1 ± 11.9^**†^
Sex, males	41 (60.3%)	58 (56.9%)	26 (65%)	6 (40%)
FEV1 (%)	87 ± 10.5	64.3 ± 10	41.4 ± 6.5	27.3 ± 4.7
FEV1/FVC (%)	67.7 ± 2.8	65.2 ± 6.2	60.3 ± 8.6	49.7 ± 16.6
Current smokers	18 (26.5%)	24 (23.5%)	12 (30%)	2 (13.3%)
Ex-smokers	40 (58.8%)	45 (44.1%)	23 (57.5%)	8 (53.3%)
Heart failure	4 (5.9%)	16 (15.7%)	17 (42.5%)^***††^	9 (60%)^***†††^
Diabetes	6 (8.8%)	22 (21.6%)*	8 (20%)	5 (33.3%)*
Hypertension	43 (63.2%)	69 (67.6%)	30 (75%)	11 (73.3%)
Cholesterol	199.8 ± 41.8	207.7 ± 40.9	190.2 ± 31.5*	191.1 ± 54.3
Body mass index	26.5 ± 3.7	26.8 ± 4.7	27.6 ± 6.0	25.9 ± 6.3

GOLD = Global Initiative for Chronic Obstructive Lung Disease; FEV1 = forced expiratory volume in the first second.

*P < 0.05, **P < 0.01, ***P < 0.001 versus GOLD stage I obtained by Student’s *t*-test for quantitative variables or chi square test for categorical variables.

^†^*P* < 0.05, ^††^*P* < 0.01, ^†††^*P* < 0.001 versus GOLD stage II obtained by Student’s *t*-test for quantitative variables or chi square test for categorical variables. FVC: Forced Vital Capacity

**Figure 2.**
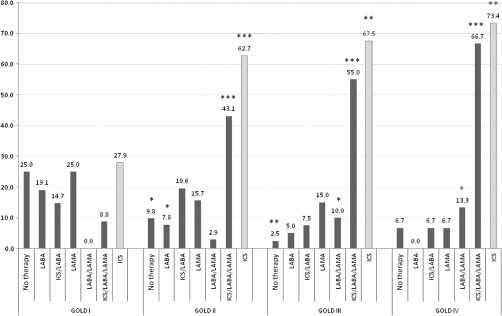
Pharmacological COPD therapy expressed as percentages in COPD patients with and without HF comorbidity, according to disease severity. *P < 0.05, **P < 0.01, ***P < 0.0001 versus GOLD stage I obtained by chi square test.

Figure 2 illustrates the medical regimens prescribed in COPD patients, with and without HF comorbidity. A comparison between patients with mild disease and patients with very severe disease shows that the percentage of patients without treatment (25% and 6.7%, *P* = 0.22), treated with LABA (19% and 0%, *P* = 0.15), LAMA (25% and 6.7%, *P* = 0.22), or with an ICS/LABA combination therapy (15% and 6.7%, *P* = 0.7) was reduced in the second group, while the percentage of patients treated with the LABA/LAMA combination (0% and 13.3%, *P* < 0.05), and even more with an ICS/LABA combined with a LAMA (9% and 67%, *P* < 0.001), increased with disease severity. The prescription of ICS increased from stage I to stage IV (27.9% in stage I, 62.7% in stage II, *P* < 0.001; 67.5% in stage III, *P* < 0.01 versus stage I; and 73.4% in stage IV, *P* < 0.01 versus stage I).

**Figure 3.**
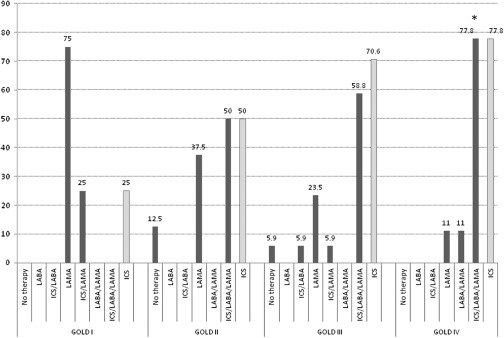
Pharmacological COPD therapy expressed as percentages in COPD patients with HF comorbidity, according to disease severity. *P < 0.05 versus GOLD stage I obtained by chi square test.

Figure 3 shows the treatment strategy in patients with COPD–HF comorbidity. The prescription of LAMA was reduced from 75% in stage I to 11% of patients in stage IV (*P* = 0.09). The LABA/LAMA combination was prescribed in stage IV only (11% of patients), while an ICS/LABA combined with a LAMA was prescribed in 50% of patients in stage II, 58.8% in stage III, and in about 78% of patients in stage IV (*P* < 0.05 in stage IV compared with stage I). Moreover, 12.5% and 6% of subjects in stages II and III, respectively, received no therapy at all.

**Figure 4.**
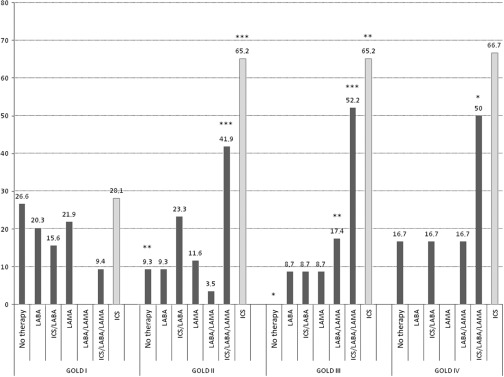
Pharmacological COPD therapy expressed as percentages in COPD patients without HF comorbidity, according to disease severity. *P < 0.05, **P < 0.01, ***P < 0.0001 versus GOLD stage I obtained by chi square test.

Figure 4 reports the pharmacological therapy in patients suffering from COPD alone. The percentage of patients treated with an ICS/LABA combined with a LAMA (9.4% and 50%, *P* < 0.05) and the percentage of those treated with a LABA/LAMA combination (3.5% and 16.7%, *P* = 0.6) increased from stage I to stage IV of disease, while the choice of LAMA was reduced (22% and 0%, *P* = 0.45).

The percentage of prescription of an ICS/LABA combination (15.6% and 16.7%) did not vary with disease severity. Furthermore, 26.6% of patients in stage I, 9.3% in stage II, and 16.7% of patients in stage IV did not receive any therapy at all.

In the LABA group, the molecules used most frequently were indacaterol, formoterol, and salmeterol. In the LAMA group, the most used molecule was tiotropium followed by glycopyrronium and aclidinium to a lesser extent. In regard to ICS, fluticasone, budesonide, and beclomethasone were the most used.

**Figure 5.**
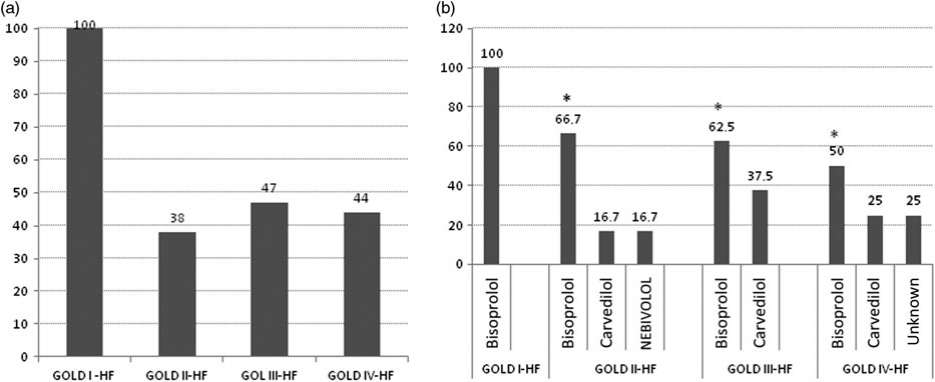
The percentage of COPD–HF patients treated with β-blockers according to COPD severity (a) and to the kind of β-blockers (b). *P < 0.05 versus GOLD stage I obtained by chi square test.

Figure 5 illustrates the percentage of patients suffering from COPD–HF comorbidity who were treated with β-blockers in relation to COPD severity (Figure 5a) and to the type of β-blockers (Figure 5b) used. Hundred percent of the patients received β-blockers in stage I, 38% in stage II, 47% in stage III, and 44% in stage IV. Bisoprolol was the most prescribed cardioselective β-blocker with a decreasing trend from stage I (100%) to stage IV (50%), *P* < 0.05.

## Discussion

Our study shows that the prevalence of HF, diabetes, and hypertension increases with severity of COPD, causing challenges with the treatment and management of these patients. We have also observed that in COPD patients, an ICS/LABA combined with a LAMA is the most prescribed treatment strategy even in the least severe stages of disease regardless of the presence of HF comorbidity. This implies that most patients with COPD are not receiving the designated treatment and have great access to high-dose ICS, which are known to increase the risk of pneumonia and carry no further benefit in terms of lung function decline and reduction in number of exacerbations per year. Conversely, the use of bronchodilators prescribed alone or in combination (LABA/LAMA), which is recommended as first-line therapy by the guidelines (Vogelmeier *et al.*, 2017b), is still limited to a small number of patients. In a previous study performed on the same cohort of patients, we found that the prevalence of HF in patients with COPD was 22% (Pirina *et al.*, 2017). These figures line up with other studies conducted on hospitalized COPD patients, where the prevalence of HF is as high as 41% (Chen *et al.*, 2015).

Conversely, the prevalence of COPD in HF patients varies between 8% and 52% as highlighted by Hawkins *et al.* (2009) in a systematic review of the literature. The same authors pointed out that in most studies, there was a significantly higher prevalence of COPD in patients with a preserved ejection fraction than in those with a reduced systolic function. A preserved ejection fraction on EcoCG may therefore delay the diagnosis of HF in patients with COPD exacerbation and, consequently hold off starting treatment for HF, which is a threat to patient safety (Stone *et al.*, 2016).

In our study, the prevalence of HF in COPD patients increased with severity of disease, ranging from 5.9% in stage I to 60% in stage IV. In addition, we found a higher prevalence of hypertension and diabetes in advanced stages of COPD.

In a follow-up study of COPD patients at eight years after diagnosis, Ställberg *et al.* (2014) reported an increase in the prevalence of HF, hypertension, and diabetes. This implies that the incidence of serious cardiovascular and metabolic comorbidities in COPD increases progressively after its first identification and along with the progression of airflow limitation, resulting in a poorer prognosis and an increased risk of death.

In 2009, Cazzola *et al.* (2009) described that only 32% of patients with a diagnosis of COPD in the general practitioners setting had undergone pulmonary function tests. Eight years on, we described that this value had reached 64%, but the remaining 36% of patients were still receiving treatment for COPD without an instrumental diagnosis of the disease. It is hence manifest that general practitioners approach the management of COPD and HF with diverging orientations, despite the similarity of these diseases in terms of prevalence, prognosis, health costs, and mortality (World Health Organization, 2016).

GOLD 2017 strategy includes the use of bronchodilators as first-choice drugs from stage I. A LABA or a LAMA or a LABA/LAMA combination can be prescribed if symptom control is not achieved. The combined therapy with LABA and LAMA has a positive effect of airflow limitation and promotes lung deflation, leading to improvement in dyspnoea, exercise tolerance, and number of exacerbations per year (Beeh and Beier, 2010; Miravitlles *et al.*, 2017b; Vogelmeier *et al.*, 2017b).

In patients with COPD–HF comorbidity, an effective pulmonary deflation leads to an improvement in cardiac performance in terms of stroke volume and left ventricular telediastolic filling (Barr *et al.*, 2010; Come *et al.*, 2012). In our study, most patients with stage I COPD were receiving a LAMA, while patients with stages II, III, or IV were being treated with ICS/LABA combination products, regardless of their HF comorbidity status. Even if these indications for bronchodilator therapy appear to be in line with the GOLD recommendations, there also appears to be an inappropriate overprescription of ICS even in the early stages of the disease. In 2012, Corrado and Rossi 2012 had already highlighted an overprescription of ICS in a large population of Italian patients. In this study, they observed that 54.6% of mild COPD, 81.8% of moderate COPD, 92% of severe COPD, and 94.8 % of very severe COPD were prescribed an ICS. Two years later, Vestbo *et al.* (2014) observed a high incidence of ICS use in a real-world population of COPD patients with Group A (39%) or B (52%) disease, which suggests that these patients were not being treated according to existing recommendations. In 2013, Yawn *et al.* (2013) assessed the association of ICS use and risk of pneumonia in a cohort of COPD patients, finding an ICS dose-related increase in risk of pneumonia. Moreover, the withdrawal of the ICS in COPD patients at low risk of exacerbations did not show either an acceleration FEV1 decline or an increase in the number of exacerbations compared to patients who continued taking ICS (Rossi *et al.*, 2014), as demonstrated in other real-life and randomised clinical trials (Magnussen *et al.*, 2014; Vogelmeier *et al.*, 2017a).

In addition to this, our study has evidenced that cardioselective β-blockers, which are first-line drugs in the treatment of HF in patients with COPD, are underprescribed, especially in advanced stages.

β-blockers are strongly recommended for patients suffering from cardiovascular diseases, particularly in HF because of their impact on mortality reduction (ACCF/AHA, 2013). They act by blocking β receptors that are expressed mainly in myocardial tissue, leading to a reduction in heart rate and myocardial contraction and therefore to a lower oxygen consumption. Two types of β-blockers have been developed: the nonselective type, which antagonizes both β 1 and β 2 receptors, and selective or cardioselective β-blockers that act mainly on β 1 receptors. One of the main concerns in the use of β-blockers in patients with COPD is that these drugs may cause worsening of lung function, already compromised by COPD. There is evidence showing that the reduction of FEV1 is significantly higher when nonselective β-blockers are preferred over the cardioselective drug. Short *et al.* (2011) conducted a study in which they examined lung function testing in 2700 COPD patients treated with β-blockers for four years. In 88% of cases, the β-blockers used were cardioselective. The authors did not find any association between the use of β-blockers and FEV1 decline in patients treated with LABA or LAMA. A meta-analysis published in 2012, which included five RCTs, showed that the mean reduction of FEV1 with the use of selective β-blockers was 30 ml, as opposed to a reduction of 140 ml when nonselective β-blockers were used (Ni *et al.*, 2012). These studies have demonstrated that cardioselective β-blockers are safe in terms of lung function impairment in patients with COPD. That being so, general practitioners should feel confident when prescribing these drugs to patients with COPD–HF comorbidity.

We think that the main strength of our research is that it is a real-life observational study which reveals how differently general practitioners approach patients with COPD and patients with COPD–HF comorbidity, based on COPD severity. We feel that this is a most interesting topic, especially if we consider that a large fraction of these patients are managed by a family doctor rather than a specialist. However, our study was limited by its retrospective nature, and we do not hold information regarding the follow-up of these patients.

## Conclusions

COPD and HF frequently coexist and this has a negative impact on prognosis, number of hospitalizations per year, healthcare costs, and mortality. This study provides new data on the management of Italian patient with COPD and HF comorbidity, highlighting that in the general practitioner setting, current treatment recommendations for COPD patients (with or without HF comorbidity), as well as guidelines for the management of HF in patients with different stages for COPD, are not being followed. We recommend general practitioners be regularly updated on guidelines and recent case–control studies regarding the safety of bronchodilators and selective β-blockers, to ensure adequate treatment for these patients with the aim to improve their survival and quality of life.
